# NLRP3 as Putative Marker of Ipilimumab-Induced Cardiotoxicity in the Presence of Hyperglycemia in Estrogen-Responsive and Triple-Negative Breast Cancer Cells

**DOI:** 10.3390/ijms21207802

**Published:** 2020-10-21

**Authors:** Vincenzo Quagliariello, Michelino De Laurentiis, Stefania Cocco, Giuseppina Rea, Annamaria Bonelli, Antonietta Caronna, Maria Cristina Lombari, Gabriele Conforti, Massimiliano Berretta, Gerardo Botti, Nicola Maurea

**Affiliations:** 1 Division of Cardiology, Istituto Nazionale Tumori- IRCCS- Fondazione G. Pascale, 80131 Napoli, Italy; a.bonelli@istitutotumori.na.it (A.B.); a.caronna@istitutotumori.na.it (A.C.); m.lombari@istitutotumori.na.it (M.C.L.); g.conforti@istitutotumori.na.it (G.C.); 2 Breast Unit, Istituto Nazionale Tumori- IRCCS- Fondazione G. Pascale, 80131 Napoli, Italy; m.delaurentiis@istitutotumori.na.it (M.D.L.); s.cocco@istitutotumori.na.it (S.C.); 3 UOC Bersagli Molecolari del Microambiente, Istituto Nazionale Tumori, IRCCS Fondazione G. Pascale, 80131 Naples, Italy; pina.rea@hotmail.it; 4 Department of MedicalOncology-Centro di Riferimento Oncologico di Aviano (CRO), IRCCS, 33081 Aviano, Italy; mberretta@gmail.com; 5 Scientific Direction, Istituto Nazionale Tumori- IRCCS- Fondazione G. Pascale, 80131 Napoli, Italy; g.botti@istitutotumori.na.it

**Keywords:** hyperglycemia, cardioncology, nivolumab, breast cancer, cytokines, cardiotoxicity

## Abstract

Hyperglycemia, obesity and metabolic syndrome are negative prognostic factors in breast cancer patients. Immune checkpoint inhibitors (ICIs) have revolutionized cancer treatment, achieving unprecedented efficacy in multiple malignancies. However, ICIs are associated with immune-related adverse events involving cardiotoxicity. We aimed to study if hyperglycemia could affect ipilimumab-induced anticancer efficacy and enhance its cardiotoxicity. Human cardiomyocytes and estrogen-responsive and triple-negative breast cancer cells (MCF-7 and MDA-MB-231 cell lines) were exposed to ipilimumab under high glucose (25 mM); low glucose (5.5 mM); high glucose and co-administration of SGLT-2 inhibitor (empagliflozin); shifting from high glucose to low glucose. Study of cell viability and the expression of new putative biomarkers of cardiotoxicity and resistance to ICIs (NLRP3, MyD88, cytokines) were quantified through ELISA (Cayman Chemical) methods. Hyperglycemia during treatment with ipilimumab increased cardiotoxicity and reduced mortality of breast cancer cells in a manner that is sensitive to NLRP3. Notably, treatment with ipilimumab and empagliflozin under high glucose or shifting from high glucose to low glucose reduced significantly the magnitude of the effects, increasing responsiveness to ipilimumab and reducing cardiotoxicity. To our knowledge, this is the first evidence that hyperglycemia exacerbates ipilimumab-induced cardiotoxicity and decreases its anticancer efficacy in MCF-7 and MDA-MB-231 cells. This study sets the stage for further tests on other breast cancer cell lines and primary cardiomyocytes and for preclinical trials in mice aimed to decrease glucose through nutritional interventions or administration of gliflozines during treatment with ipilimumab.

## 1. Introduction

Immune checkpoint inhibitors (ICIs) improved overall survival in cancer patients both as monotherapy or combined with chemotherapies for primary and metastatic cancer patients [1,2]. The family of ICIs involves anti-PD-1 (called nivolumab and pembrolizumab), anti-PD-L1 (called atezolizumab, avelumab, and durvalumab) and anti-CTLA-4 antibodies (called ipilimumab and tremelimumab) [1,3,4]. The combinatorial strategies of ICIs are currently under study in metastatic cancer patients, aimed to reduce immune-resistance of cancer cells, enhancing their apoptosis and necrosis [5]. However, ICIs showed several autoimmune or inflammatory side effects, collectively termed immune-related adverse events, including diabetes, chronic inflammatory bowel diseases, thyroiditis and cardiovascular diseases [6]. Cardiovascular immune-related adverse events involved myocarditis [7]; its pathogenesis is based on lymphocytic infiltration in myocardial tissue and a direct/indirect interaction with cardiomyocytes expressing PD-1/PDL-1 and other immune-sensitive antigens [7,8]. The prevalence of myocarditis ranged from 0.06% to 2.4%, with a higher risk in combination immunotherapy [8]. Other cardiovascular diseases induced by ICIs involves pericardial disease, vasculitis, Takotsubo syndrome, destabilization of atherosclerotic lesions, venous thromboembolism, and conduction abnormalities [9]. Patients with diabetes have increased risk of breast, liver, bladder, pancreatic, colorectal, endometrial and prostate cancers [10,11]. Hyperglycemia is a well-recognized prognostic factor in patients with several chronic diseases like myocardial injuries and cancer [12,13]. Hyperglycemia increases the prevalence and mortality of cancer patients [14]. Causes of cancer risks in diabetic patients involved hyperglycemia, hyperinsulinemia, insulin resistance, distorted insulin-like growth factor-1 (IGF-1) pathway, oxidative stress, enhanced inflammatory processes and aberrant sex hormone production [15,16]. Joshi et al. [17] pointed out that hyperglycemia could provide nutrients for the rapid proliferation of malignant tumor cells, thereby accelerating the process of tumor cells. Hou et al. [18] reported that high-concentration glucose (25 mM) significantly increased the proliferation of breast cancer cells compared to low-concentration glucose (5 mM). Hyperglycemia accelerates the progression of tumor, increases the proliferation, migration and invasion of cancer cells [19]. Recent findings reported that hyperglycemia could increase anticancer-induced cardiotoxicity [20] through involvement of AMPK, mitochondrial proteins, reactive oxygen species and pro-inflammatory cytokines involved in pro-fibrogenic and pro-apoptotic signaling [21,22].

NLRP3 is a new prognostic marker in oncology and acts as a key player in immune-related events involving bacterial and viral infection as well as autoimmune diseases [23,24]. NLRP3 inflammasome activation increases gene expression of IL-1 and IL-6, thereby enhancing production of hs-CRP [23]. Recently, NLRP3 inflammasome was proposed as a new biomarker of cardiovascular diseases and predictor of hospitalization and death for myocardial injuries [25,26]. MyD88 complex (called myddosome) is another protein regulator of death-like signals in human cells [27]; it is a putative marker of the incidence and prognosis of cardiovascular diseases and cancer [28]. However, to the best of our knowledge, no studies have analyzed the effects of high glucose or low glucose on breast cancer responsiveness to ICIs and their cardiotoxicity. To date, only few studies correlated NLRP3 and MyD88 with hyperglycemia damages, like diabetes-induced endothelial inflammation and atherosclerosis [26]. Considering the high prevalence of breast cancer in women with diabetes [29,30], we studied if hyperglycemia could exacerbate ipilimumab-induced cardiotoxicity and decreases its anticancer efficacy in human breast cancer cells (estrogen responsive and triple negative cells) and verified the involvement of NLRP3 and MyD88 in these processes. Moreover, we highlighted the effects of the low glucose or Sodium glucose co-transporter 2 inhibitor (SGLT-2i), called empagliflozin, on the reduction of magnitude of the pro-inflammatory effects mediated by hyperglycemia on cancer cells and cardiomyocytes.

## 2. Results

### 2.1. CTLA-4 Expression in Human Breast Cancer Cells

**Figure 1:**
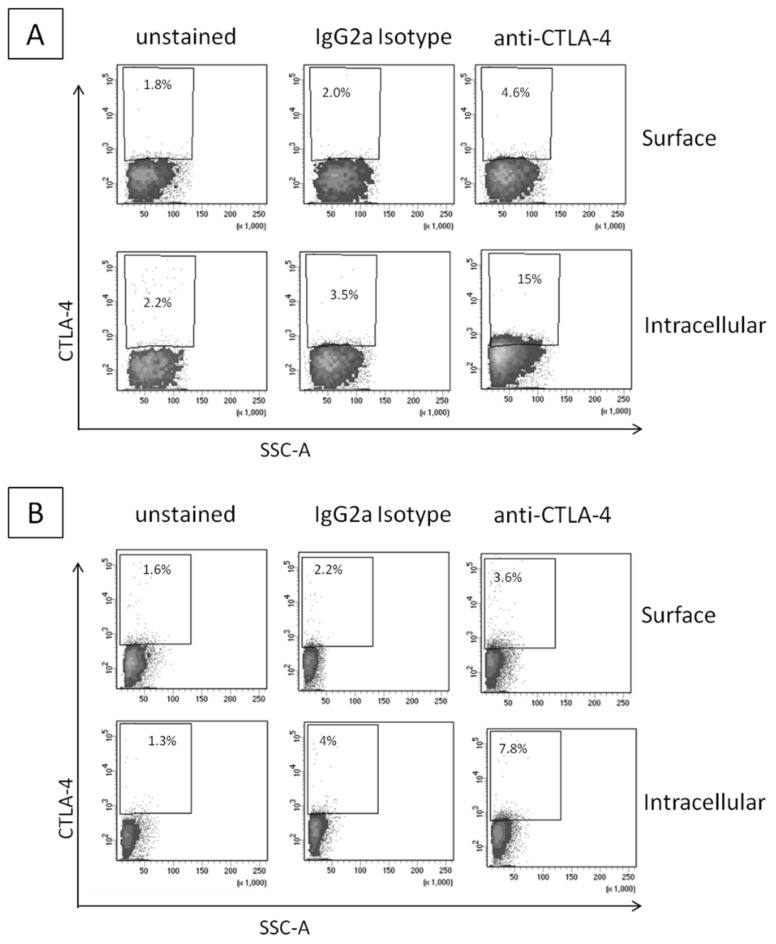
Flow-cytometric analysis of CTLA-4 in human breast cancer cells MDA-MB-231 (**A**) and MCF-7 (**B**). MDA-MB-231 and MCF-7 were stained on their surface or intracellularly with the designated antibodies. IgG2a Isotype corresponds to the staining with a negative class-matched control antibody. Results are expressed as percentage of stained cells.

Firstly, we investigated the intracellular and surface expression of CTLA-4 in breast cancer cell lines by Fluorescence-activated cell sorting (FACS) analysis. As expected and reported in the literature, CTLA-4 expression in the breast cancer cell lines was detectable; the higher expression was seen in MDA-MB-231 cells (Figure 1A) compared to MCF-7 (Figure 1B). Moreover, the intracellular expression was generally higher than the surface expression and these data are in line with the literature, confirming the interesting putative role of CTLA-4-related pathway in the breast cancer cell metabolism.

### 2.2. Glucose Reduces Ipilimumab-Related Anticancer Activities and Increases its Cardiotoxicity

**Figure 2:**
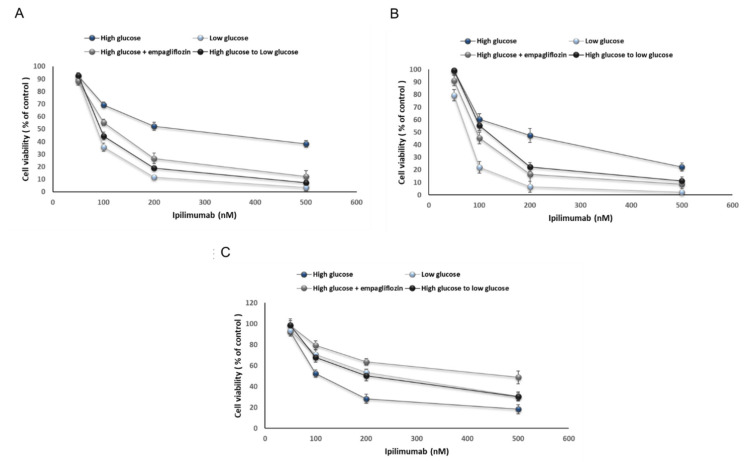
Cell viability of MCF-7 (**A**) and MDA-MB-231 (**B**) cells after 72 h of incubation with ipilimumab under different condition (high glucose; low glucose; high glucose + empagliflozin at 500 nM; switch high glucose to low glucose); (**C**) Cell viability of AC16 cells after 72 h of incubation with ipilimumab under different condition (high glucose; low glucose; high glucose + empagliflozin at 500 nM; shifting from a high glucose to low glucose). Error bars depict means ± SD (*n* = 3). Statistical analysis was performed using paired *t*-test.

We studied how high glucose could affect the anticancer properties and cardiotoxicity induced by ipilimumab in a co-culture of cardiomyocytes or breast cancer cells with human peripheral blood mononuclear cells (hPBMCs) [19]. We found that sensitivity to ipilimumab was reduced by 25 mM glucose compared to 5.5 mM glucose (Figure 2A,B). Shifting from a high glucose to a low glucose as well as the treatment with empagliflozin ameliorated breast cancer cell responsiveness to ipilimumab (Figure 2A,B for MCF-7 and MDA-MB-231 cells, respectively). Notably, triple negative breast cancer cells showed more sensitivity to ipilimumab than MCF-7 cells. A different behavior was seen in cardiomyocytes co-cultured with PBMCs (Figure 2C): hyperglicemia increased significantly ipilimumab-induced toxicity than hypoglicemia (paired *t*-test *p* < 0.001, *n* = 3); administration of empagliflozin during high glucose and shifting from high glucose to low glucose reduced the magnitude of the effects. These results indicated that hyperglicemia significantly influenced the cytotoxicity of ipilimumab in breast cancer cells and cardiomyocytes; low glucose and exposure to empagliflozin under hyperglicemia increases the anticancer efficacy of the CTLA-4 blocking agent in breast cancer cells and reduces cytotoxicity.

### 2.3. Glucose Increases Leukotriene-Mediated Cardiotoxicity of Ipilimumab

**Figure 3:**
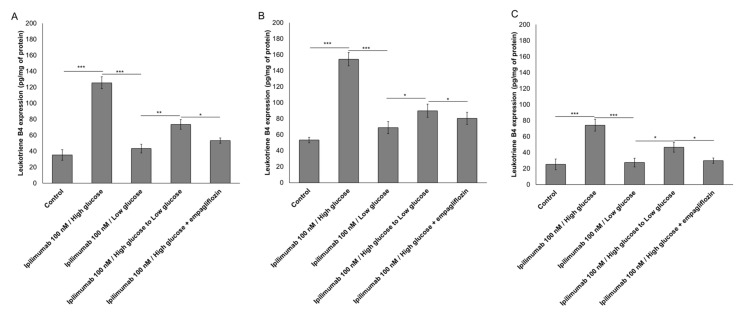
Leukotrienes type B4 production by MCF-7 (**A**) and MDA-MB-231 (**B**) cells, treated with ipilimumab mAb for 24 h, in the presence of human peripheral blood mononuclear cells (hPBMCs) under different condition (high glucose; low glucose; high glucose + empagliflozin at 50 nm; shifting from a high glucose to low glucose). Untreated or treated cells with an unrelated control IgG (control) were used as negative controls; (**C**) Leukotrienes type B4 production by AC-16 cells, treated with ipilimumab mAb for 24 h, in the presence of hPBMCs under different condition (high glucose; low glucose; high glucose + empagliflozin at 500 nM; shifting from a high glucose to low glucose). Untreated or treated cells with an unrelated control IgG (control) were used as negative controls. Error bars depict means ± SD (*n* = 3). Statistical analysis was performed using paired *t*-test. *** *p* < 0.001.** *p* < 0.01.* *p* < 0.05.

To evaluate the effects of hyperglicemia on lipid metabolism transduction signal pathways during ipilimumab exposure in cancer cells and cardiomyocytes, we quantified the production of leukotrienes B4 (Figure 3). After incubation with ipilimumab under hyperglicemia, MCF-7 cells increased production of leukotrienes compared to low-glucose (125.6 ± 7.4 vs. 43,3 ± 5.5 pg/mg of protein, paired *t*-test *p* < 0.001, *n* = 3) (Figure 3A); shifting from high glucose to low glucose (73.5 ± 6.1 vs. 125.6 ± 7.4 pg/mg of protein, paired *t*-test *p* < 0.001, *n* = 3), as well as the treatment with empagliflozin under hyperglicemic conditions (53.3 ± 3.3 vs. 125.6 ± 7.4 pg/mg of protein, paired *t*-test *p* < 0.001, *n* = 3) reduced significantly the production of leukotrienes indicating anti-inflammatory effects (Figure 3A). A different picture was seen in MDA-MB-231 cells (Figure 3B); after incubation with ipilimumab under hyperglicemia, triple negative cells increased production of leukotrienes compared to low-glucose (154.5 ± 8.3 vs. 53,6 ± 3.4 pg/mg of protein, paired *t*-test *p* < 0.001, *n* = 3) (Figure 3A); shifting from high glucose to low glucose (89.9 ± 8.2 vs. 154.5 ± 8.3 pg/mg of protein, paired *t*-test *p* < 0.001, *n* = 3), as well as the treatment with empagliflozin under hyperglicemic condition (80.5 ± 7.6 vs. 154.5 ± 8.3 pg/mg of protein, paired *t*-test *p* < 0.001, *n* = 3) reduced significantly the production of leukotrienes indicating anti-inflammatory effects (Figure 3B). Human cardiomyocytes exposed to ipilimumab under hyperglicemic conditions (74.2 ± 7.4 vs. 27.2 ±5.4 pg/mg of protein, paired *t*-test *p* < 0.001, *n* = 3) increased the production of leukotrienes and these effects were partially reduced after a change to low-glucose (46.6 ± 6.1 pg/mg of protein) and treatment with empagliflozin (29.9 ± 3.3 pg/mg of protein) (Figure 2B).

### 2.4. Hyperglycemia Have Pro-Oxidative Effects during Treatment with Ipilimumab

**Figure 4:**
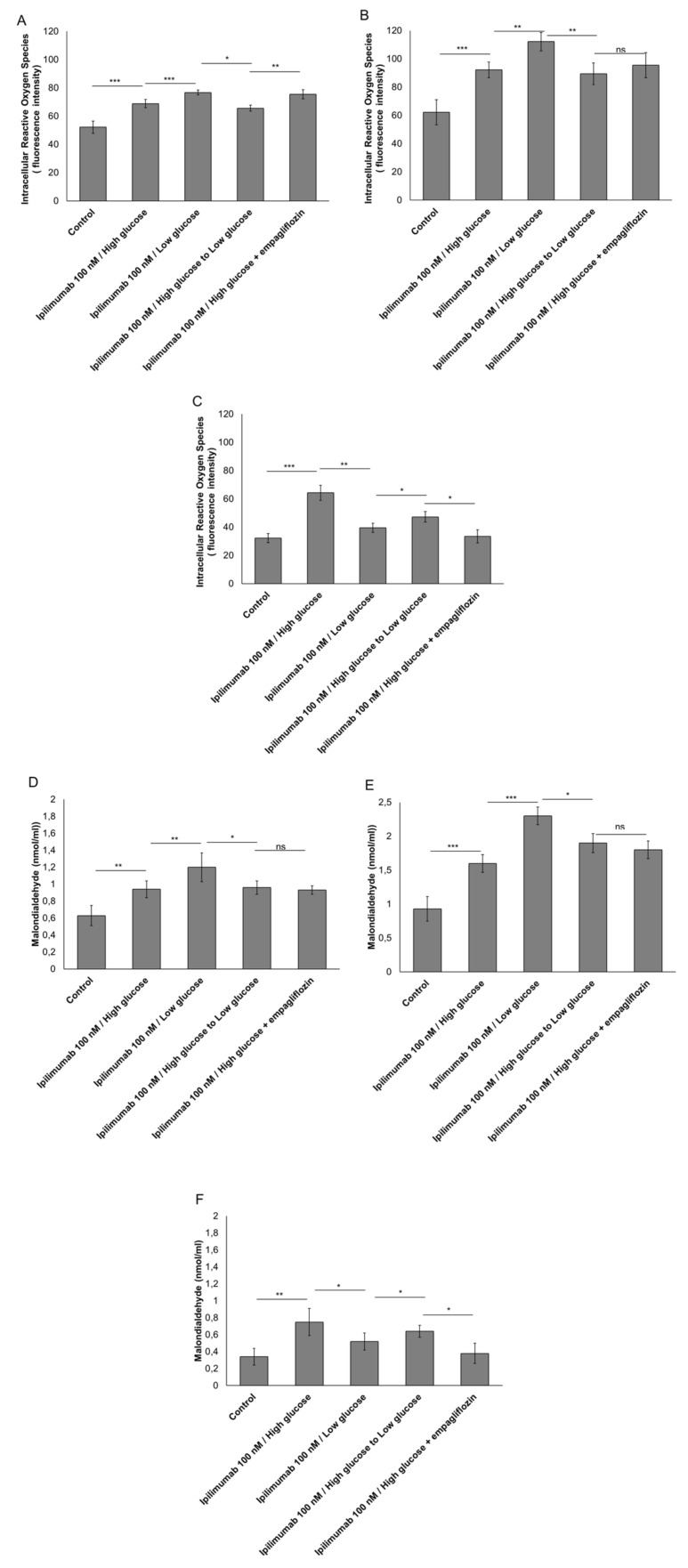
Intracellular Reactive Oxygen Species (ROS) and Malondialdeyde (MDA) quantification in MCF-7 cells (**A**,**D**) and MDA-MB-231 (**B**,**E**) cells treated with ipilimumab mAb in the presence of hPBMCs under different condition (high glucose; low glucose; high glucose + empagliflozin at 50 nm; shifting from a high glucose to low glucose). Untreated or treated cells with an unrelated control IgG (control) were used as negative controls; (**C**,**F**) Intracellular Reactive Oxygen Species (ROS) and Malondialdeyde (MDA) quantification in AC-16 cells, treated with ipilimumab mAb for 24 h, in the presence of hPBMCs under different conditions (high glucose; low glucose; high glucose + empagliflozin at 500 nM; shifting from a high glucose to low glucose). Untreated or treated cells with an unrelated control IgG (control) were used as negative controls. Error bars depict means ± SD (*n* = 3). Statistical analysis was performed using paired *t*-test. *** *p* < 0.001. ** *p* < 0.01. * *p* < 0.05.

Reactive oxygen species (ROS) overproduction induces several cellular damages and activates pro-inflammatory pathways in myocytes and cancer cells [31,32]. In breast cancer cells, incubation with ipilimumab increased ROS production compared to untreated cells (Figure 4A,B). Under low glucose or after treatment with empagliflozin, surprisingly, ROS production was increased (Figure 4A,B). A different picture was seen in cardiomyocytes (Figure 4C); in fact, treatment with ipilimumab under low glucose or during empagliflozin partially reduced ROS production compared with myocytes grown under hyperglicemic conditions (Figure 4C). These effects were confirmed through the quantification of malondialdeyde (MDA) as a marker of lipid peroxidation [33] that was increased significantly in MCF-7 (Figure 4D) and MDA-MB-231 cells (Figure 4E) and reduced in cardyomyocytes (Figure 4F) under low glucose.

### 2.5. p65-NF-κB is Overexpressed under Hyperglicemic Condition

**Figure 5:**
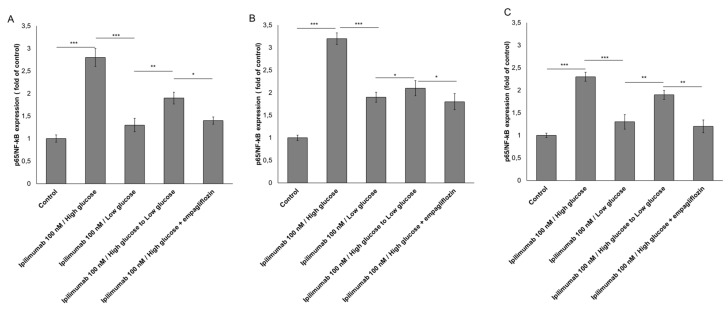
p65/NF-kB expression(fold of control) expression in MCF-7 (**A**), MDA-MB-231 (**B**) and AC-16 (**C**) cells, treated with ipilimumab mAb in the presence of hPBMCs under different condition (high glucose; low glucose; high glucose + empagliflozin at 500 nM; shifting from a high glucose to low glucose). Untreated or treated cells with an unrelated control IgG (control) were used as negative controls. Error bars depict means ± SD (*n* = 3). Statistical analysis was performed using paired *t*-test. *** *p* < 0.001. ** *p* < 0.01. * *p* < 0.05.

As shown in Figure 5A,B, p65-NF-κB expression was increased by 2.8 and 3.4 times in MCF-7 and MDA-MB-231 cells, respectively, under high glucose and exposure to ipilimumab. This trend was reduced by shifting high glucose to low glucose (−0.9 ± 0.13 for MCF-7 cells; −1.2 ± 0.11 for MDA-MB-231 cells; paired *t*-test *p* < 0.001, *n* = 3 for both) and after administration of empagliflozin (−1.4 ± 0.008 for MCF-7 cells; −1.6 ± 0.03 for MDA-MB-231 cells; paired *t*-test *p* < 0.001, *n* = 3 for both). Additionally, cardiomyocytes exposed to ipilimumab under high glucose increased by 2.3 times the expression of p65-NF-κB compared with untreated cells and shifting from high glucose to low glucose reduced the magnitude of the effects (Figure 5C). These effects indicate anti-inflammatory properties of hypoglicemia and treatment with empagliflozin during treatment with ipilimumab.

### 2.6. NLRP3 and MYD88 Expression Are Key Mediators of Hyperglicemia-Mediated Effects in Human Breast Cancer Cells and Cardiomyocytes

**Figure 6:**
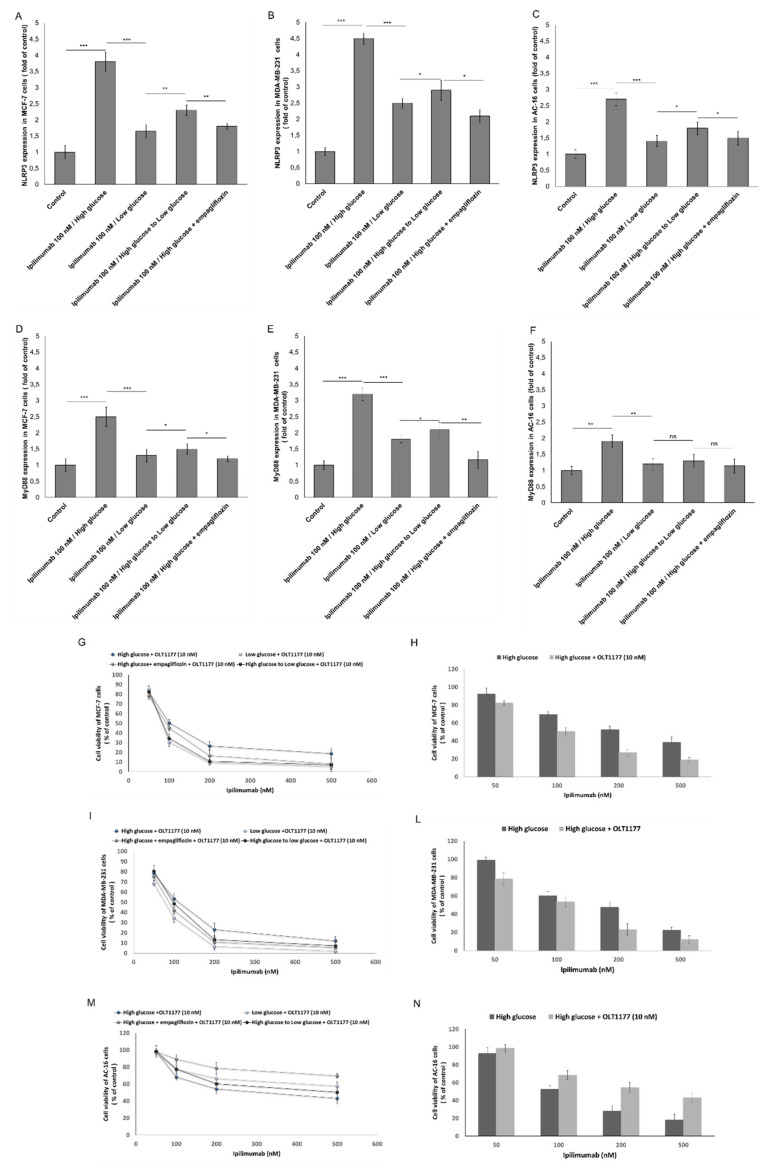
NLRP3 (fold of control) expression in MCF-7 (**A**), MDA-MB-231 (**B**), and AC-16 cells (**C**), treated with ipilimumab mAb in the presence of hPBMCs under different condition (high glucose; low glucose; high glucose + empagliflozin at 500 nM; shifting from a high glucose to low glucose). MyD88 (fold of control) expression in MCF-7 (**D**), MDA-MB-231 (**E**), and AC-16 cells (**F**), treated with ipilimumab in the presence of hPBMCs under different condition (high glucose; low glucose; high glucose + empagliflozin at 50 nm; shifting from a high glucose to low glucose). Cell viability of MCF-7 (**G**,**H**), MDA-MB-231 (**I**,**L**), and AC-16 cells (**M**,**N**) under high glucose (with or without empagliflozin), low glucose, shifting from high glucose to low glucose and always exposed to ipilimumab and NLRP3 selective inhibitor OLT-1177. For all experiments, untreated or treated cells with an unrelated control IgG (control) were used as negative controls. Error bars depict means ± SD (*n* = 3). Statistical analysis was performed using paired *t*-test. *** *p* < 0.001. ** *p* < 0.01. * *p* < 0.05.

We investigated on NLRP3 and MyD88 as key prognostic factors of dendrimental effects of hyperglicema in ipilimumab-induced cardiotoxicity and anticancer effects. In MCF-7 cells, NLRP3 (3.8 ± 0.3 vs. 1.65 ± 0.2, (fold of control) paired *t*-test *p* < 0.001, *n* = 3) (Figure 6A) and MyD88 (2.5 ± 0.3 vs. 1.3 ± 0.1, (fold of control) paired *t*-test *p* < 0.001, *n* = 3) (Figure 6D) were clearly overexpressed under high glucose compared with low glucose. In MDA-MB-231 cells, NLRP3 (4.5 ± 0.14 vs. 2.4 ± 0.11, (fold of control) paired *t*-test *p* < 0.001, *n* = 3) (Figure 6B) and MyD88 (3.3 ± 0.21 vs. 1.7 ± 0.12, (fold of control) paired *t*-test *p* < 0.001, *n* = 3) (Figure 6E) were clearly overexpressed under high glucose compared with low glucose. These effects were reversible by shifting from high glucose to low glucose (Figure 6). Lower levels of NLRP3 and MyD88 protein in high glucose cells treated with empagliflozin were also seen (Figure 6). To assess if NLRP3 controls the sensitivity of high glucose cells to ipilimumab, MCF-7 (Figure 6G,H) and MDA-MB-231 cells (Figure 6I,L) were treated with the anti CTLA-4 antibody in the presence (or absence) of OLT1177 (a selective NLRP3 inhibitor). Treatment with OLT1177 significantly increased responsiveness to ipilimumab in both hyperglicemic and hypoglicemic conditions (Figure 6). A similar behavior was seen in cardiomyocytes: hyperglycemia increased expression of NLRP3 and MyD88 in a way that is sensitive to empagliflozin or shifting from high glucose to low glucose (Figure 6C,F). Selective inhibition of NLRP3 decreases significantly the cardiotoxicity of ipilimumab under high glucose (Figure 6M,N).

### 2.7. NLRP3 Staining in Breast Cancer Cells and Cardiomyocytes

**Figure 7:**
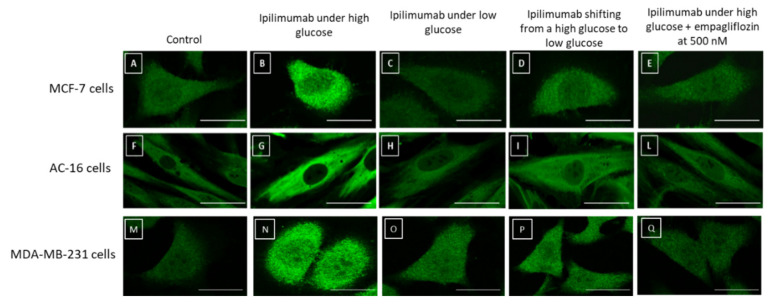
NLRP3 straining (green signals) in MCF-7 (**A**–**E**), AC-16 (**F**–**I**,**L**), and MDA-MB-231 cells (**M**–**Q**) treated with ipilimumab under high glucose (**B**,**G**,**N**); low glucose (**C**,**H**,**O**); shifting from a high glucose to low glucose (**D**,**I**,**P**), and high glucose + empagliflozin at 500 nM (**E**,**L**,**Q**). Untreated cells with an unrelated control IgG (control) were used as negative controls (**A**,**F**,**M**). Scale bar: 50 µm.

NLRP3 plays a key role in the pathogenesis of breast cancer and heart failure. Based on the considerable changes in NLRP3 expression in MCF-7, MDA-MB-231, and AC-16 cells under high glucose and low glucose, we analyzed cellular staining of NLRP3 through a confocal laser scanning microscope (Figure 7). Breast cancer cells (Figure 7B,N) and cardiomyocytes (Figure 7G) under high glucose and exposed to ipilimumab showed a considerably higher amount of NLRP3 (green signals) than the control (Figure 7A,F,M). Treatment with empagliflozin under high glucose (Figure 7E,L,Q), shifting from high glucose to low glucose (Figure 7D,I,P) and growth in low glucose (Figure 7C,H,O), decreased significantly NLRP3 staining in cell cytoplasm indicating anti-inflammatory effects. These results corroborated the quantitative data described in Figure 6.

### 2.8. Pro-Inflamamtory Cytokines and Growth Factors Are Overexpressed during Hyperglycemia

**Figure 8:**
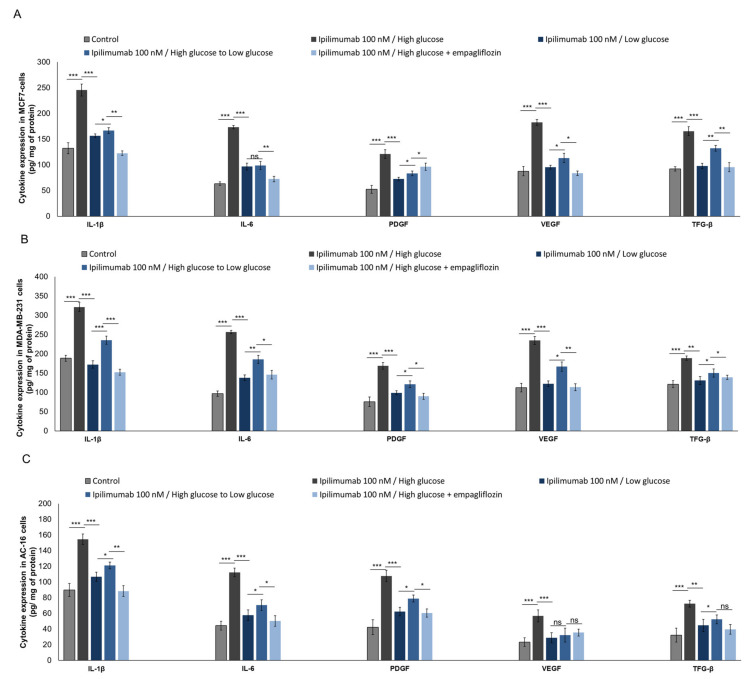
Expression of IL-1, IL-6, PDGF, VEGF, and TGF in MCF-7 (**A**), MDA-MB-231 (**B**), and AC-16 cells (**C**). Cells were treated with ipilimumab mAb for 24 h, in the presence of hPBMCs under different condition (high glucose; low glucose; shifting from a high glucose to low glucose; high glucose + empagliflozin at 500 nm). Error bars depict means ± SD (*n* = 3). Statistical analysis was performed using paired *t*-test. *** *p* < 0.001. ** *p* < 0.01. * *p* < 0.05.

NLRP3 and MyD88 are primary activators of cytokine storm in human cells in response to pro-inflammatory stimuli, as well as bacterial and viral infection [34]. We investigated cytokines and growth factors during exposure to ipilimumab under hyperglycemia, hypoglycemia, exposure to empagliflozin (under high glucose), and after shifting from high glucose to low glucose. Effectively, under hyperglycemia, compared to low glucose, MCF-7 cells exposed to ipilimumab (Figure 8A) overexpressed IL-1β (245.5 ± 11.5 vs. 156.6 ± 3.4 pg/mg of protein, paired *t*-test *p* < 0.001, *n* = 3), IL-6 (173.3 ± 3.3 vs. 96.6 ± 6.7 pg/mg of protein, paired *t*-test *p* < 0.001, *n* = 3), PDGF (121.1 ± 8.3 vs. 72.3 ± 3.5 pg/mg of protein, paired *t*-test *p* < 0.001, *n* = 3), VEGF (182.5 ± 5.5 vs. 95.3 ± 3.8 pg/mg of protein, paired *t*-test *p* < 0.001, *n* = 3), and TGF-β (165.5 ± 8.5 vs. 97.7 ± 5.3 pg/mg of protein, paired *t*-test *p* < 0.001 *n* = 3). A slightly different picture was seen for triple negative breast cancer cells (Figure 8B): under hyperglycemia, compared to low glucose, MDA-MB-231 cells exposed to ipilimumab overexpressed IL-1β (321.1 ± 10.8 vs. 188.4 ± 7.6 pg/mg of protein, paired *t*-test *p* < 0.001, *n* = 3), IL-6 (256.6 ± 4.5 vs. 102.8 ± 7.2 pg/mg of protein, paired *t*-test *p* < 0.001, *n* = 3), PDGF (168.8 ± 8.9 vs. 78.9 ± 12.2 pg/mg of protein, paired *t*-test *p* < 0.001, *n* = 3), VEGF (234.4 ± 10.3 vs. 112.5 ± 11.2 pg/mg of protein, paired *t*-test *p* < 0.001, *n* = 3), and TGF-β (188.8 ± 5.6 vs. 121.1 ± 8.9 pg/mg of protein, paired *t*-test *p* < 0.001 *n* = 3). For both, shifting from high glucose to low glucose or being treated with empagliflozin under high glucose reduced significantly the expression of cytokines and growth factors compared with cells exposed to high glucose (Figure 8A,B). Instead, cardiomyocytes exposed to ipilimumab under high glucose (Figure 8C) overexpressed IL-1β (154.4 ± 6.7 vs. 106.6 ± 5.8 pg/mg of protein, paired *t*-test *p* < 0.001, *n* = 3), IL-6 (112.2 ± 5.6 vs. 57.7 ± 6.7 pg/mg of protein, paired *t*-test *p* < 0.001, *n* = 3), PDGF (107.7 ± 7.2 vs. 62.1 ± 5.5 pg/mg of protein, paired *t*-test *p* < 0.001, *n* = 3), VEGF (56.7 ± 7.7 vs. 28.7 ± 6.4 pg/mg of protein, paired *t*-test *p* < 0.001, *n* = 3), and TGF-β (72.3 ± 4.3 vs. 44.7 ± 7.8 pg/mg of protein, paired *t*-test *p* < 0.001, *n* = 3), compared with low glucose cells. Also in this case, under low glucose or after treatment with empagliflozin, the increase rates of cytokines and growth factors were significantly reduced.

## 3. Discussion

In this study, we demonstrated that hyperglycemia reduces ipilimumab-related anticancer functions and enhances its cardiotoxicity in cellular models through mechanisms mediated by MyD88 and NLRP3 signaling. More specifically, our findings provide a proof of principle that hyperglycemia increased cytokine storm in human breast cancer cells and cardiomyocytes placing the conditions for cardiotoxic and immune-resistance phenomena. Several studies demonstrated that hyperglycemia or diabetes increased both incidence and recurrence of cancer (breast, liver, prostate, brain, and pancreas) [35]. Chronic or intermittent hyperglycemia is associated with the development of diabetic complications like cardiovascular diseases and chronic inflammatory diseases. Several signaling pathways could be modified during high glucose, for example the increase of inoxidative stress and the overproduction of advanced glycation end products (AGEs) associated with pro-inflammatory cytokines leading to cellular death or chemo-resistance [36,37]. However, the signaling pathways directly triggered by hyperglycemia appear to have a pivotal role in diabetic complications due to the production of reactive oxygen species (ROS) and lipid peroxides [38]. However, surprisingly, in co-cultures of breast cancer cells and hPBMCs, low glucose increases ROS production (Figure 3) compared to high glucose; these effects could be explained by the production of ROS following the cytotoxic effect of hPBMCs against cancer cells [39]; T-cell mediated cytotoxicity involves granzyme B and other pro-apoptotic and pro-oxidizing factors. [39] However, here we have not studied the type of cell death induced by high glucose or the amount of granzyme B secreted by hPBMCs. Further studies on the cell death mechanism will be performed.

Hyperglycemia and Amadori products induce the overexpression of ERK/MAPK leading to the production of ROS and pro-inflammatory and immune-modulating interleukins like interleukin-1 [40]. Interleukin 1 is a key factor in the progression of cancer, cardiovascular diseases, and mortality [41]. Recent cardiovascular outcome trials (CVOTs) demonstrated that pharmacological inhibition of interleukin-1 by subcutaneous administration of the anti IL-1 antibody (i.e., canakimumab) reduced the risk of adverse cardiac events and mortality [42]. Other studies showed that patients treated with canakimumab had reduced cancer-related mortality compared with untreated patients [43]. Results of this study focus on a direct link between hyperglycemia and the pro-inflammatory cytokines/growth factors involved in cancer survival and cardiotoxicity during the CTLA4 blocking agent ipilimumab; these effects are reversible by shifting from high glucose to low glucose or through concomitant treatment with an anti SGLT-2 drug (empagliflozin). Recent studies in cancer-bearing mice demonstrated the chemo-preventive and anti-inflammatory properties of calorie-restriction [44]. In another study, low glucose was associated with higher response to tamoxifen in breast cancer patients [19]. Clinical trials on calorie restriction or hypoglycemia and responsiveness to ICIs are scarce: one retrospective cohort study performed in 55 cancer patients demonstrated trending improvements in overall and progression-free survival in participants with metastatic malignant melanoma who receive a hypoglycemic drug (metformin) in combination with ipilimumab/nivolumab and/or pembrolizumab [45].

Another trial is currently under investigation to combine metformin with ICIs in non-small cell lung cancer patients (NCT03048500) [46]. Other trials in non-cancer [47] and cancer [48,49] patients suggests that natural flavonoids with hypoglycemic properties (i.e., resveratrol) improve T-cell function and increase responsiveness to anti-cancer drugs. Clearly, there is a need for larger retrospective analyses and multi-center prospective studies aimed to evaluate the potential benefits anti-hyperglycemic agents or calorie restriction mimetic strategies (such as hydroxycitrate, metformin, and complementary and alternative medicines) combined with ICIs or conventional anticancer drugs [50,51,52].

MyD88 is a molecular complex involved in regulation of the immune system, cardiovascular, and cancer metabolism [27]. Patients with viral myocarditis have a high expression of MyD88, CD3+ lymphocytes, and collagen fibers (markers of fibrosis) in myocardial tissue [27]. MyD88 is essential for stimulating resistance to paclitaxel, doxorubicin, and tamoxifen [28]; however, its roles in the ICI-related resistance of breast cancer cells remain unclear. Here, we hypothesize that MyD88, directly associated with hyperglycemia, could be directly involved in the cardiotoxicity and anticancer functions of ipilimumab. Notably, shifting from hyperglycemia to hypoglycemia, as well as the treatment with empagliflozin, reduced the magnitude of these effects and expression of MyD88 both in MCF7, MDA-MB-231 cells, and AC16 cells.

Inflammasomes are multiprotein complexes regulating pro-inflammatory factors including IL-1βand IL-18. IL-18 induces programmed cell death protein 1-dependent immunosuppression in cancer [53], and IL-1β is one of the most important pro-inflammatory mediators involved in immune resistance [54]. The nucleotide-binding oligomerization domain-like receptors (NOD-like receptors) family pyrin domain, containing 3 (NLRP3), is the most widely studied inflammasome. A previous study demonstrated that activated NLRP3 increases IL-18 in patients with lymphoma [55]. NLRP3 inflammasome could represent a novel potential target for the treatment of breast cancer [56]. In a recent preclinical study, the pharmacological inhibition of NLRP3 through miRNA reduced the tumor growth and the immune-resistance in breast cancer-bearing mice through ASC/IL-1/IL-18 pathways; these data provide new clinical insights for breast cancer management [57]. Notably, NLRP3 is associated with myocardial injuries, atherosclerosis, and diabetes mellitus [57]; high glucose stimulates NLRP3 expression thorough inhibition of its ubiquitinization in human cells [57]; therefore, we can speculate that hyperglycemia could enhance cardiotoxicity and responsiveness to ipilimumab through the NLRP3 complex. Therefore, NLRP3 could became a predictive marker of immune-resistance and cardiotoxicity to ipilimumab. Notably, high glucose breast cancer cells and cardiomyocytes exposed to ipilimumab increased NLRP3 inflammasome expression. Administration of the NLRP3 selective inhibitor (OLT1177) increased responsiveness to ipilimumab in breast cancer cells and reduced cytotoxicity in AC16 cells. Interleukin-6 is another key player in ICI-induced cardiotoxicity; it is an independent predictor of diabetes and cardiovascular diseases [58]. Adipocytes and macrophages are the major sources of IL-6 in patients with metabolic syndrome and obesity [58]. Notably, IL-6 has immunosuppressive properties in colorectal cancer cells through the recruitment of immune-suppressive cells and reduction of T cell infiltration in cancer tissues [59]. Inhibition of IL-6 enhanced the efficacy of anti-PD-L1 antibodies in colorectal cancer providing a novel strategy to overcome anti-PD-L1 resistance [60]. Here, we have shown that high glucose increased IL-6 and IL-1 expression in breast cancer cells and cardiomyocytes exposed to ipilimumab; notably, also in this case, shifting from high glucose to low glucose and the treatment with empagliflozin reduced significantly IL-6 expression, providing new insight on the putative protective role of low glucose in IL-6 mediated immune-suppression and cardiotoxicity.

Based on this scenario, overexpression of NLRP3/MyD88 and cytokines during hyperglycemia in breast cancer cells and cardiomyocytes could be a key player in cardiotoxicity and resistance to ipilimumab. There are some limitations of this study: firstly, human leukocyte antigen (HLA)-class I molecules on tumor cells have been regarded as crucial sites where cytotoxic T lymphocytes can recognize tumor-specific antigens. HLA mismatch could be a possible limitation of co-cultures of tumor cells with hPMBCs, despite co-culture models often being used and recommended in preliminary cell-lymphocytes interaction studies, as reported in the literature [61,62,63,64]. Although many immortalized cell lines may have a reduced HLA expression [65,66,67], it is conceivable that some cancer cells induce an HLA mismatch when co-incubated with hPBMCs, being from different donors. However, in line with other similar papers published in the literature [61,62,63,64], for all tested combinations (in our case controls and treatments with ipilimumab under high glucose and low glucose) the differences between groups are still due to the treatment conditions. Moreover, different open discussion papers are available about the role, for example, of HLA-E in co-cultures of cancer cells and PBMCs [68]. Interestingly, a recent study Tristan Courau et al. [68] points to another resistance mechanism used by tumor cells that try to evade immune recognition. This is illustrated by HLA-E upregulation on tumor cells upon spheroid infiltration, associated with NKG2A increase in infiltrating immune cells. NKG2A-HLA-E pathway has already been described as a potential inhibitor of antitumor immune responses. A deep study of the role of high glucose in HLA-E expression in co-cultures of breast cancer cells and PBMCs should be also investigated. Another limitation of the study is the lack of a deeper analysis of metabolome in breast cancer cells and cardiomyocytes under high glucose or low glucose. Other studies will be performed during treatments with other ICIs, including anti-PD-1 and anti PDL-1 blocking agents. Moreover, preclinical studies will be made on mice models following a hyperglycemic/hypoglycemic diet, in order to corroborate cellular results described herein. The effects seen herein are principally due to the effects of ipilimumab on hPBMCs and not against cancer cells or cardiomyocytes. To date, the mechanisms and key players of ipilimumab-induced myocardial injuries are not completely understood [69]. Immune cells uptake and infiltration in heart tissues were always seen in human histological studies with high amounts of CD4+/CD8+ T lymphocytes and CD68+ cells [70]; this interaction involves some chemokines like Interleukin 1, 6, and 8 and chemokines that increase the granzyme B-mediated cytotoxicity driving cardiac injury [71]. Treatment with ipilmumab and other ICIs increase the cancer cell recognition of lymphocytes and their release of cytotoxic degranulation markers perforin and granzyme B [72]. According to the literature, CTLA-4 is also expressed in a subsample of breast cancer cells and their role in cancer cell responsiveness to ICIs is actually unknown [73]. It is possible that isolated CTLA-4 positive breast cancer cells could have a different responsiveness to ipilimumab or hyperglycemia; further studies will be performed on isolated CTLA-4 + breast cancer cells, studying their metabolism and their responsiveness to glucose and growth factors.

High glucose mediates NLRP3 inflammasome activation via upregulation of E74-like transcription factor (ELF3) expression [74]. Microtubule affinity regulating kinase 4 (MARK4) plays a crucial role in the regulation of NLRP3 inflammasome activation, which leads to the generation of IL-1β [74]. High glucose increases NLRP3 activation, probably involved in ICIs-induced resistance and cardiotoxicity; in fact, recently, a mechanism was identified whereby CD8+ T cell activation in response to PD-1 blockade induced a NLRP3 inflammasome signaling cascade that ultimately led to the recruitment of granulocytic myeloid-derived suppressor cells (MDSCs) into tumor tissues, thereby dampening the resulting antitumor immune response [75]. Genetic and pharmacologic inhibition of NLRP3 enhances the efficacy of anti–PD-1 Ab immunotherapy [76]. We hypothesize that high glucose increases NLRP3 expression thereby reducing the ipilimumab-mediated cytotoxic efficacy against MCF-7 and MDA-MB-231 cells. To our knowledge, this is the first evidence that hyperglycemia exacerbates ipilimumab-induced cardiotoxicity and decreases its anticancer efficacy in MCF-7 and MDA-MB-231 cells. Moreover, to clarify whether hypoglycemia can increase responsiveness to ipilimumab against ER+ and triple negative breast cancer and reduces cardiovascular side effects, further studies in breast cancer-bearing mice are also required.

## 4. Materials and Methods

### 4.1. Cell Culture

AC16 human cardiomyocytes were purchased from American Type Culture Collection (ATCC^®^, LGC Standards, Teddington, UK) and cultured in Gibco^®^ Dulbecco’s modified Eagle’s medium (Gibco, Milan, Italy): Nutrient mixture F-12 (DMEM/F12) supplemented with 10% fetal bovine serum (FBS) (HyClone™, GE Healthcare Life Sciences, Milan, Italy) and Penicillin-Streptomycin (100 U/mL, Gibco^®^, Milan, Italy). MCF-7 human breast cancer cells (ERα+, PR+, HER2-) were cultured in Dulbecco’s modified Eagle’s medium (DMEM) supplemented with 10% fetal bovine serum (FBS), 2 mM glutamine, 100 units/mL penicillin and 100 units/mL streptomycin. Triple negative MDA-MB-231 (ATCC^®^ HTB-26™) cells were grown in ATCC-formulated Leibovitz’s L-15 Medium supplemented with 10% fetal bovine serum (FBS) (HyClone™, GE Healthcare Life Sciences, Milan, Italy) and Penic illin-Streptomycin (100 U/mL, Gibco^®^, Milan, Italy). Cell cultures were maintained in a humidified atmosphere of 95% air and 5% CO_2_ at 37 °C.

### 4.2. Co-Cultures

To test the biological effects of hyperglycemia on breast cancer and cardiac metabolism, we used co-cultures of MCF-7 and MDA-MB-231 breast cancer cells or human cardiomyocytes (AC16) (that do not express CTLA-4) with human peripheral blood mononuclear cells (hPBMCs). Cancer cells and cardiomyocytes were plated in 96-well flat-bottom plates at the density of 150,000 cells/well for 16 h. hPBMCs, a population of immune cells consisting of T cells, B cells, natural killer cells, dendritic cells, and monocytes [77] from healthy donors, were added at effector: target ratio 5:1 in the absence or presence of ipilimumab as described previously [78,79]; in fact, hPBMCs are conventionally used in cellular experiments of responsiveness to PD-1, PDL-1, or CTLA-4 blocking agents [80,81,82]. Co-culture of MCF7 or MDA-MB-231 cells or AC16 cells with hPBMCs mimics, respectively, the immune cell infiltration in breast cancer tissue (the immune infiltration of tumors is closely related to clinical outcomes in breast cancer patients [83,84]), as well as in myocardial tissue (patients treated with CTLA-4 blocking agents developed severe/fatal myocarditis which was likely a result of the lymphocytic infiltration of lymphocytes [85,86]).

### 4.3. CTLA-4 Expression in Breast Cancer Cells through Flow Cytometry

For the cell-surface marker, MDA-MB-231 and MCF-7 cells (100.000 cells/tube) were harvested and stained with anti-human CD152 (CTLA-4) (BD Bioscience, San Diego, CA, USA) and LIVE/DEAD fixable Aqua (Thermo Fisher, Milan, Italy, at 4 °C for 30 min in cell stain Buffer (BSA 0.2%) (BD Pharmingen™, San Diego, CA, USA). Cells were then washed twice and resuspended in 200 μL of in cell stain Buffer. To avoid non-specific anti-CTLA-4 antibody binding to the Fc portion of the receptor, the cell suspension was pre-treated with FcR Blocking Reagent, human (130-059-901 MiltenyiBiotec, GmbH, Bergisch Gladbach, Germany), and then stained with the anti-CTLA-4 antibody. As negative control, we stained cells with a negative class-matched control antibody (IgG2a isotype). For intracellular staining, cells were fixed and permeabilized using intracellular buffer set BD Cytofix/Cytoperm™ (BD Bioscience, San Jose, CA, USA) according to the manufacturer’s instructions and stained with anti-human CD152 at ice for 30 min in permeabilization buffer. The samples were washed and resuspended in 200 μL of cell stain Buffer (BSA0.2%) (BD Pharmingen™, San Diego, CA, USA) cells were washed twice with permeabilization buffer. A minimum of 100.000 events for each sample were collected by FACS ARIA III (Becton Dickinson, Mountain View, CA, USA) and data were analyzed using FACSDiva™ 8.0 Software (BD Bioscience, San Diego, CA, USA).

### 4.4. Cell Viability

To test the effects of hyperglycemia on cellular mitochondrial viability, we work with co-cultures of MCF-7 or MDA-MB-231 cells or human cardiomyocytes (AC16) with hPBMCs, the cells were plated in 96-well flat-bottom plates at the density of 150,000 cells/well for 16 h. Human Peripheral Blood Mononuclear Cells (hPBMCs) were added at an effector:target ratio of 5:1 in the absence or presence of ipilimumab at 50, 100, 200 and 500 nM and incubated for 72 h at 37 °C, as described previously [78,79]. Controls included target cells incubated in the absence of effector cells or in the presence of anti CTLA-4 antibody. Notably, cells in co-culture were grown in 5.5 mM glucose, corresponding to normal fasting glucose levels in humans, or in 25 mM glucose resembling hyperglycemia in humans, following well established protocols [19]; moreover, as a control, only during the hyperglycemic condition, cells were co-incubated with ipilimumab (100 nM, [78]) and empagliflozin (500 nM, [87]), an oral antidiabetic agent with cardioprotective properties. After treatments, lymphocytes were removed and adherent cells were washed three times with PBS at pH 7.4 and incubated with 100 μL of an MTT solution (0.5 mg/mL in cell culture medium) for 4 h at 37 °C. Absorbance readings were acquired at a wavelength of 450 nm with the Tecan Infinite M200 plate-reader (Tecan Life Sciences Home, Männedorf, Switzerland) using I-control software. Relative cell viability (%) was calculated with the following formula: [A]test/[A]control × 100, where “[A]test” is the absorbance of the test sample, and “[A]control” is the absorbance of the control cells incubated solely in culture medium. After the evaluation of cell cytotoxicity, we measured the total protein content using the Pierce Micro BCA protein assay kit (Thermo Fisher, Milan, Italy). Briefly, the cells were washed with ice-cold PBS, and incubated for 15 min in 150 μL cell lysis buffer (0.5% *v*/*v* Triton X-100 in PBS) that included 150 μL of the Micro BCA protein assay kit reagent (prepared according to the manufacturer’s instructions). Absorbance at 562 nm was measured on a plate reader. Cytotoxicity measurements were normalized by the amount of total protein content in each well.

### 4.5. Expression of Leukotriene B4 (LTB4)

Co-cultures of cardiomyocytes/hPBMCs and breast cancer cells/hPBMCs were untreated (control) or treated with ipilimumab (100 nM) under high glucose, low glucose, shifting from high glucose to low glucose, or treated with empagliflozin (500 nM) under high glucose for 12 h. After treatments, leukotriene B4 ((5S,12R)-dihydroxy-6,14Z-8,10E-eicosatetraenoic acid) expression in cell lysates was determined through ELISA (Cayman Chemical, Ann Arbor, MI, USA) following the supplier’s instructions [78]; data were expressed as pg of leukotriene B4/mg of cell proteins calculated by QuantiPro Assay (Biorad, Milan, Italy).

### 4.6. Reactive Oxygen Species and Lipid Peroxidation

Lipid peroxidation is a key player in heart failure and chemo-resistance phenomena. Co-cultures of cardiomyocytes/hPBMCs and breast cancer cells/hPBMCs were untreated (control) or treated with ipilimumab (100 nM) for 6 h under high glucose, low glucose, shifting from high glucose to low glucose, or treated with empagliflozin (500 nM) under high glucose. After treatments, cells were washed three times with cold PBS, harvested with 0.25% *v*/*v* Trypsin, and centrifuged at 1000× *g* for 10 min. The supernatant was discarded and the cell pellet sonicated in cold PBS. After a centrifugation step at 800× *g* for 5 min, we quantified malondialdehyde (MDA) by using a commercial kit with a spectrophotometer according to the manufacturer’s protocols (Sigma Aldrich, Milan, Italy) [79]. We measured the protein content of the cell homogenates using the Micro BCA protein assay kit (Pierce, Thermo Fisher, Milan, Italy) according to the kit instructions.

### 4.7. p65-NF-κB Expression

Co-cultures of cardiomyocytes/hPBMCs and breast cancer cells/hPBMCs were untreated (control) or treated with ipilimumab (100 nM) under high glucose, low glucose, shifting from high glucose to low glucose or treated with empagliflozin (500 nM) under high glucose for 12 h. After treatments, cells were harvested and lysed in lyses buffer (50 mMTris-HCl, pH 7.4, 1 mM EDTA, 100 mM NaCl, 20 mM NaF, 3 mM Na_3_VO_4_, 1 mM PMSF, and protease inhibitor cocktail). Lysates were then centrifuged, the supernatants were collected and analyzed using the TransAM NF-κB p65 transcription factor assay kit (Active Motif, Carlsbad, CA, USA), according to the manufacturer’s recommendations. NF-κB complexes were captured by binding to a consensus 5′-GGGACTTTCC-3′ oligonucleotide immobilized on a 96-well plate. Bound NF-κB was quantified by incubating with anti-p65 primary antibody followed by horseradish peroxidase (HRP)-conjugated goat anti-rabbit IgG and spectrophotometric detection at a wavelength of 450 nm using a microplate spectrofluorometer. Data were expressed as the percentage of p65/NF-κB DNA binding relative to control (untreated) cells.

### 4.8. NLRP3 and MyD88: Key Mediators of Hyperglycemia-Induced Cardiotoxicity

Co-cultures of cardiomyocytes/hPBMCs and breast cancer cells/hPBMCs were untreated (control) or treated with ipilimumab (100 nM) under high glucose, low glucose, shifting from high glucose to low glucose, or treated with empagliflozin (500 nM) under high glucose for 12 h. After treatments, cells were harvested and lysed in lyses buffer (50 mMTris-HCl, pH 7.4, 1 mM EDTA, 100 mM NaCl, 20 mM NaF, 3mM Na_3_VO_4_, 1 mM PMSF, and protease inhibitor cocktail). Lysates were then centrifuged, the supernatants were collected and submitted to the ELISA protocol for quantification of MyD88 (Human MyD88 ELISA Kit (ab171341), Abcam, Milan, Italy) and NLRP3 (Human NLRP3 ELISA Kit (OKEH03368), Aviva Systems Biology, San Diego, CA, USA) [88,89]. Briefly, an antibody against NLRP3 or MyD88 was pre-coated onto a 96-wellplate (12 × 8 Well Strips) and blocked. Standards or test samples were added to the wells and incubated for 1h. After washing, a biotinylated detector antibody specific to NLRP3 or MyD88 was added, incubated and followed by washing for 30 s. Avidin-Peroxidase Conjugate was then added, incubated, and unbound conjugate was washed away. An enzymatic reaction was produced through the addition of TMB substrate which is catalyzed by HRP generating a blue color product that changes yellow after adding acidic stop solution. The density of yellow coloration read by absorbance at 450 nm was quantitatively proportional to the amount of sample NLRP3 or MYD88 captured in well. For human MyD88 ELISA, the sensitivity was <10 pg/mL and range of detection was 156 pg/mL–10,000 pg/mL; for human NLRP3 ELISA assay, the sensitivity was <0.078 ng/mL and range of detection was 0.156–10 ng/mL. Moreover, we utilized pharmacological inhibitor to identify the major signaling pathway involved in hyperglicemia-related cancer sensitivity and the cardiotoxicity of ipilimumab. To this aim, we pre-incubated MCF-7, MDA-MB.231, and AC-16 cells with NLRP3 inhibitor (OLT1177) at 100 nM [90] during incubation with ipilimumab and performed cell viability assay as described in paragraph 4.4.

### 4.9. Confocal Laser Scanning Microscope

Cardiomyocytes and human breast cancer cells cultured as described previously under standard conditions at 37 °C in a humidified 5% CO_2_ atmosphere, were seeded in a 24-well plate (5000 cells/well) and allowed to grow for 24 h. Cells were then untreated (control) or treated with ipilimumab (100 nM) under high glucose, low glucose, shifting from high glucose to low glucose, or treated with empagliflozin (500 nM) under high glucose for 12 h. Cells were then fixed with 2.5% glutaraldehyde in PBS at room temperature for 20 min, rinsed with PBS, and permeabilzed with 0.1% Triton X-100 for 5 min. NLRP3 was stained by incubation with a primary antibody against NLRP3 (Life Span Bio Sciences) for 1 h under gentle stirring, followed by an anti-human NLRP3 polyclonal antibody (Life Span BioSciences, Seattle, WA, USA). The detection was performed by the addition of a Goat Anti-Rabbit IgG H&L (FITC) (ab6717, Abcam, Milan, Italy) for 1 h, under gentle stirring. Using a Confocal Microscope (C1 Nikon) equipped with a EZ-C1 Software for data acquisition and 60× oil immersion objective, NLRP3 was imaged through excitation/emission at 488/515 nm.

#### 4.9.1. Cytokines and Growth Factors Assay

The expression of IL-1β, IL-6, PDGF, FGF, VEGF, and TGF-βin cardiomyocytes and breast cancer cells was performed through the ELISA method, as described elsewhere [78]. Co-cultures of cardiomyocytes/hPBMCs and breast cancer cells/hPBMCs were untreated (control) or treated with ipilimumab (100 nM) under high glucose, low glucose, shifting from high glucose to low glucose, or treated with empagliflozin (500 nM) under high glucose for 12 h. Culture supernatants were centrifuged to pellet any detached cells and measured using the appropriate ELISA kits according to the manufacturer’s instructions (Sigma Aldrich, Milan, Italy). The sensitivity of this method was below 10 (pg/mL), and the assay accurately detected cytokines in the range of 1–32,000 pg/mL.

#### 4.9.2. Statistical Analysis

All cell-based assays were performed in triplicates (*n* = 3) and results are presented as mean ± Standard Deviation (SD). To compare cell culture conditions, a paired-*t* test was used through the use of Sigmaplot software (Systat Software Inc., San Jose, CA, USA). *p* < 0.05 was considered to indicate a statistically significant difference.

## 5. Conclusions

Hyperglycemia is a prognostic marker in oncology; glucose-related damages increased cancer cell metabolism, chemo, and immune resistance [91]; in fact, integrating glycometabolism targeting (aimed to reduce glucose in cancer cells) and immunotherapy seems to be a rational strategy for improving overall survival in cancer patients [92]. This study reveals that hyperglycemia during treatment with ipilimumab, a CTLA-4 blocking agent, increased cardiotoxicity and reduced mortality of MCF-7 and MDA-MB-231 cells in a manner that is sensitive to NLRP3. Therefore, NLRP3 could became a valid biomarker of ipilimumab-induced cardiotoxicity under hypoglycemia; pharmacological inhibition of NLRP3, through clinically available drugs [90,93], safe in humans, currently studied for therapy of acute gout and arthritis could be an effective therapeutic strategy aimed at improving anticancer responsiveness to ipilimumab and reducing its cardiovascular side effects. Notably, cells treated with ipilimumab and SGLT-2 inhibitor (empagliflozin) under high glucose or shifting from high glucose to low glucose reduced significantly the magnitude of the effects. Further studies will be done in other breast cancer cell lines and cardiomyocytes (i.e., primary ventricular cardiomyocytes) exposed to CTLA-4 blocking agents as well as other immune check point inhibitors (PD-1 or PDL-1 blocking agents). This study set the stage to preclinical trials in mice models aimed to decrease glucose through nutritional intervention or through treatment with gliflozines during therapy with ipilimumab.

## Abbreviations

ICIs	Immune checkpoint inhibitors
SGLT-2	Sodium-glucose cotransporter type-2
NLRP3	NOD-like receptor family pyrin domain, containing 3
MyD88	Myddosome type 88
PD1	Programmed cell death protein 1
PDL1	Programmed Death-Ligand 1
CTLA4	Cytotoxic T-Lymphocyte Antigen 4
AMPK	5’ AMP-activated protein kinase
hs-CRP	High sensitivity C-reactive protein
ROS	Reactive oxygen species
MDA	Malondialdeyde
p65-NF-κB	Nuclear factor kappa-light-chain-enhancer of activated B cells
ILs	Interleukins
PDGF	Platelet-Derived Growth Factor
VEGF	Vascular endothelia growth factor
TGF-β	Transforming growth factor beta
AGEs	Advanced glycation end products
CVOTs	Cardiovascular outcome trials
VEGF	Vascular endothelia growth factor
TGF-β	Transforming growth factor beta
AGEs	Advanced glycation end products
